# AgNPs Targeting the Drug Resistance Problem of *Staphylococcus aureus*: Susceptibility to Antibiotics and Efflux Effect

**DOI:** 10.3390/pharmaceutics14040763

**Published:** 2022-03-31

**Authors:** Ekaterina Nefedova, Nikolay Shkil, Roberto Luna Vazquez-Gomez, Diana Garibo, Alexey Pestryakov, Nina Bogdanchikova

**Affiliations:** 1 Siberian Federal Scientific Centre of Agro-BioTechnologies of the Russian Academy of Sciences, Novosibirsk 630501, Russia; filll555@mail.ru (E.N.); nicola07@mail.ru (N.S.); 2 Escuela de Ciencias de la Salud, Universidad Autónoma de Baja California, Ensenada 22890, B.C., Mexico; 3 Centro de Nanociencias y Nanotecnología, Universidad Nacional Autónoma de México, Ensenada 22800, B.C., Mexico; dgaribo@conacyt.mx; 4 Consejo Nacional de Ciencia y Tecnología, Ciudad de México 03940, Mexico; 5 Research School of Chemistry and Applied Biomedical Sciences, Tomsk Polytechnic University, Tomsk 634050, Russia; pestryakov2005@yandex.ru

**Keywords:** AgNPs, lactobay, *S. aureus*, efflux effect

## Abstract

The present work presents translational research with application of AgNPs targeting the global drug resistance problem. In vivo fieldwork was carried out with 400 breeding farm cows sick with a serous mastitis. Ex vivo results revealed that after cow treatment with Lactobay^TM^ (a mixture of antibiotic drugs) the susceptibility to 31 antibiotics of *S. aureus* isolates from cow breast secretion decreased by 25%, while after treatment with Argovit–C^TM^ silver nanoparticles *S. aureus* susceptibility increased by 11%. The portion of isolates with an efflux effect leading to elimination of antibiotics from *S. aureus* after Lactobay-treatment resulted in a 15% increase, while Argovit-C-treatment led to a 17.5% decrease. The obtained results showed that mastitis treatments with Argovit-C^TM^ AgNPs can partially restore the activity of antibiotics towards *S. aureus* and shorten the duration of mastitis treatment by 33%.

## 1. Introduction

Drug-resistant bacteria are evolving pathogens with strong resistance profiles and their spread becomes difficult to contain, generating a negative impact on human, plant, and animal health. Multidrug resistance has become a growing global problem in the treatment of infectious diseases caused by life-threatening infections. It is estimated that 4.95 million deaths were associated with bacterial antimicrobial resistance (AMR), including 1.27 million deaths attributable to bacterial AMR [1]. The AMR causes high economic losses in the health sector. For example, in Europe the overall crude economic burden of AMR was estimated to be at least EUR 1.5 billion with more than EUR 900 million corresponding to hospital costs [2] and in the USA USD 55 billion per year [3]. Thus, due to the health implications and economic losses, the World Health organization (WHO) considered the antibiotic resistance as the major priority for developing an urgent action to contain the AMR [4].

To control bacterial AMR several alternative methods were proposed. Some of them are the use of essential oils, antibodies, bacteriocins and phage therapy. These types of compounds showed antimicrobial activity alone and in combination with antibiotics (synergistic effect). Application of these compounds is promising, but they still have disadvantages: low storage stability, high prices and laborious and time-consuming methods of their synthesis that require highly qualified personnel [5]. Therefore, the world needs alternative, cost-effective and efficient antimicrobial agents capable once more to kill multi-resistant bacterial species. Nowadays, nanomaterials have achieved remarkable attention as novel antimicrobial products. Silver nanoparticles (AgNPs) are the most studied among them [6]. AgNPs alone or in combination with antibiotics (synergy) inhibit the growth of microorganisms, including resistant bacterial strains [7].

In 2017, the WHO published global priority pathogens list designated by the acronym ESKAPE (*Enterococcus faecium*, *Staphylococcus aureus*, *Klebsiella pneumoniae*, *Acinetobacter baumannii*, *Psudomonas aeruginosa* and *Enterobacter* species). The highest “priority status” was given to ESKAPE species because they represent an exceptional threat to humans [8]. There are four mechanisms of antibiotic resistance developed in ESKAPE pathogens: decreased drug uptake, drug target alteration, drug inactivation, and drug efflux pumps activation [8,9,10].

The capacity of AgNPs to inhibit efflux pump activity was revealed recently. In a review published in 2017, dedicated to nanoparticles as efflux pump inhibitor (EPIs) [11], nothing has been mentioned about AgNPs. The first mention of AgNPs as EPIs we found in a 2018 publication, in which obtained results only provided circumstantial (not direct) evidence indicative of AgNPs functioning as EPIs [12]. For the last three years, the articles published results of in vitro experiments showing that AgNPs can decrease the efflux pump gene expression, reduce expression of functional efflux pump proteins, inhibit activity of P-glycoprotein (Pgp, the drug transporters that determine the uptake and efflux of drugs), or change efflux pump activity [12,13,14,15,16,17,18,19,20,21,22]. These results were obtained with application of AgNPs alone or in combination with antibiotics or other drugs, to which microorganisms or cells had already developed resistance. These publications include influence of AgNPs on the efflux effect of *P. aeruginosa* [13,14,15], *Escherichia coli* [17,18,19], *A. baumannii* [14,15,16], *K. pneumonia* [20,21], *Burkholderia pseudomallei* [22], *E. cloacae* [12]. Moreover, the influence of AgNPs on the efflux effect was studied on *Bifidobacterium bifidum* probiotic, the drug-resistant cancer MCF-7/KCR cell line [23], and planktonic cells and biofilms [24]. *K. pneumoniae*, *A. baumannii* and *P. aeruginosa* are three bacteria from six species in WHO’s global priority pathogens list. However, we did not find the data concerning AgNPs as EPIs for *S. aureus* which is also in WHO’s global priority pathogens list.

Recent publications present results of in vitro research on the efflux effect produced by AgNPs or AgNPs in combination with one or two drugs. The results of in vitro research are elucidating the role of AgNPs as active efflux pump inhibitors and opens the perspective to translate (convert) this knowledge to practical application in combating the AMR that will directly serve a human benefit. This work represents the continuation of our previous work [25] and is dedicated studying the influence of AgNPs on the efflux effect of *S. aureus*, which is one of the six WHO global priority pathogens, not studied before, under conditions of a livestock farm. According to our best knowledge, this is the first large-scale translational study, where the efflux effect of *S. aureus* isolates of 400 cows was studied in relation to 31 conventional antibiotics before and after treatments with AgNPs or Lactobay.

## 2. Experimental

**Figure 1:**
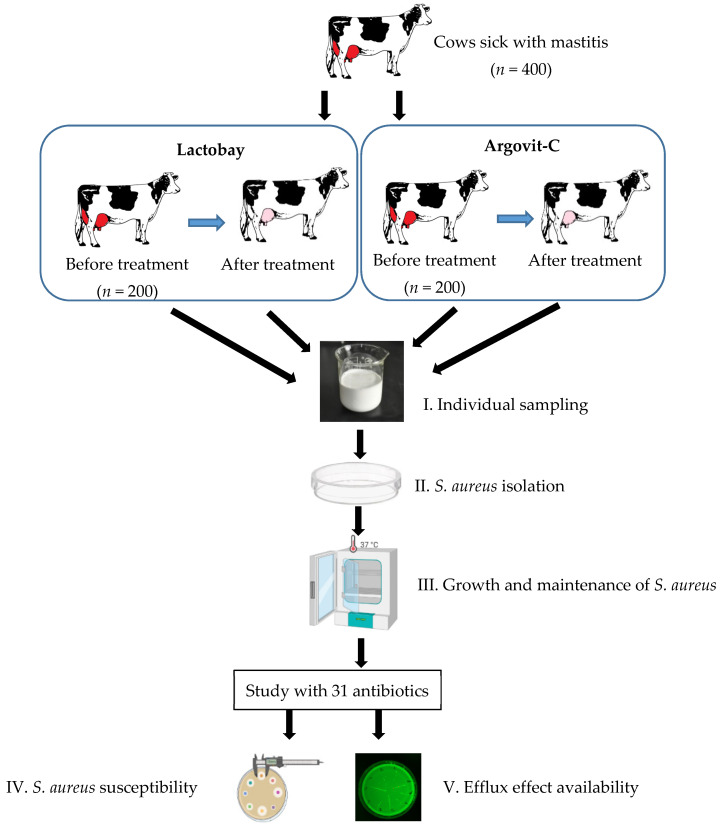
Experimental design diagram. Sampling (I), *S. aureus* isolation (II), *S. aureus* propagation (III), subsequent studies of antibiotic susceptibility (IV) and efflux effect (V) were carried out before and after treatments with Lactobay or Argovit-C.

The present work was carried out with 400 breeding farm cows sick with a serous mastitis. The diagnosis was made by clinical symptoms followed by confirmation with the Biochemical California test [26]. Complete recovery was established also on the basis of the Biochemical California test, which was undertaken every day during treatment. The cows were divided into two equal groups of 200 cows each (Figure 1). The first group involved cows treated with Lactobay, which is first-line veterinary drug for mastitis treatment. The second group included 200 cows treated with Argovit-C silver nanoparticles. Samples of cow breast secretion (milk) from both groups were analyzed before and after their intracisternal treatment with one of the two formulations mentioned (after complete recovery). Samples taken before the treatments can be considered as reference. The experimental design diagram is shown in Figure 1.

### 2.1. Sampling

Milk samples were taken from cows with mastitis before and after treatment with Lactobay or Argovit-C under conditions of livestock farms of the Novosibirsk, Russian Federation region during 2018–2019 (stage I in Figure 1). For sampling, the udder teats were wiped with a cotton swab moistened with 70% ethyl alcohol. Milk in a volume of 10 mL was collected into clean test tubes avoiding contact of the nipple with the edge of the test tubes. A test tube with milk was closed with a cotton-gauze stopper and the name or inventory number of the cow was written on the tube label. The samples were stored at a temperature of 8–10 °C until testing. Within 3–4 h, the samples were delivered for research.

### 2.2. Treatment Formulations

Lactobay^TM^, Norbrook Laboratories Limited, Newry, Northern Ireland, UK, is an antibacterial drug for intracisternal administration in the form of a suspension, containing two antibiotics (1.5% sodium ampicillin and 4% sodium cloxacillin) as active ingredients. The drug Lactobay in veterinary medicine is used as a first-line drug for the treatment of mastitis. The cows were injected intracisternally with Lactobay in a dose of 5 g with an interval of 12 h in accordance with the instructions of the manufacturer. The treatment was administered within 6 days until complete recovery (justified with the Biochemical California test).

Argovit-C^TM^ produced by Vector-Vita Scientific and Production Center, Novosibirsk, Russia, was provided by Dr. Vasily Burmistrov. Argovit-C, used for therapeutic and prophylactic purposes in the case of gastrointestinal diseases of calves, is a stable water suspension of silver nanoparticles (AgNPs) with a concentration of 200 mg/mL (20 wt.%). The metallic silver concentration is 12 mg/mL (1.2 wt.%) and silver particle size is in the interval of 5–20 nm with an average diameter of 15 nm. AgNPs are stabilized by polivinilpirrolidone and hydrolyzed collagen with a total concentration of 18.8 wt.%. The remaining 80% of the weight corresponds to distilled water. Argovit-C was administered intracisternally to animals of the experimental group with serous form of mastitis (*n* = 200) after 10-fold Argovit-C dilution (equivalent to 1.2 mg/mL of metallic Ag) at a dose of 10 mL once a day for 4 days until complete recovery, justified with the Biochemical California test.

### 2.3. Isolation and Identification of S. aureus Bacteria

The material for the study was the secretion of the mammary gland of animals, namely the bacteria *S. aureus*, isolated from secretion before and after cow treatment with Lactobay or Argovit-C formulations (stage II in Figure 1). Isolation of staphylococci was performed using a selective additive Staph-Strepto Supplement (HiMedia Laboratories Pvt. Ltd., Mumbai, India). Colonies of staphylococci were identified using the *Staphylococcus* 24 test (Erba Lachema s.r.o., Brno, Czech Republic). The identification at the species level of the microbiota isolated from animals was carried out, taking into account the cultural, morphological, and biochemical properties of bacteria according to the generally accepted methods described in “The Bergey’s manual of determinative bacteriology”, 2000 [27].

### 2.4. Antimicrobial Susceptibility and Efflux Testing

Microorganism susceptibility was determined by disc diffusion method on Mueller–Hinton agar (Bio-Rad, Hercules, CA, USA), in accordance with the criterion of The Clinical and Laboratory Standards Institute (CLSI) [28].

The study of the efflux effect consisted in streaking the culture of *S. aureus* microorganisms on Eugonic agar with ethidium bromide (1 mg/L). After 24 h of cultivation, the detection of efflux was carried out using a transilluminator [29]. In the absence of the efflux effect, ethidium bromide penetrates into the bacterial cell and its complex with DNA glows in ultraviolet rays, while when efflux occurs no fluorescence is detected.

Studies on susceptibility and the efflux effect (stages IV and V in Figure 1) of *S. aureus* isolated from the milk of cows, sick with serous mastitis, were carried out ex vivo for 31 antibiotics belonging to 8 groups: 1. Aminoglycosides (amikacin, neomycin, streptomycin, kanamycin, gentamicin, tobramycin); 2. Fluoroquinolones (ciprofloxacin, enrofloxacin, norfloxacin, ofloxacin); 3. Tetracyclines (tetracycline, doxycycline); 4. Penicillins (carbenicillin, ampicillin, oxacillin, benzylpenicillin (penicillin), amoxicillin); 5. Cephalosporins (cephalexin, cefuroxime, cefotaxime, ceftiofur, cefazolin, ceftazidime); 6. Macrolides (erythromycin, tylosin) and lincosamides (lincomycin); 7. Nitrofurans (furagin, furazolidone); 8. Others (polymyxin, rifampicin, levomycetin (chloramphenicol)).

The susceptibility and efflux effect studies were undertaken before and after treatments with drugs of different pharmacological groups (Argovit-C and Lactobay).

### 2.5. Statistical Analysis

The results were statistically processed using the methods of parametric and nonparametric analysis. The accumulation, correction, systematization of the initial information, and visualization of the results were carried out with GraphPad Software 9.0, San Diego, CA, USA. Statistical analysis was carried out using the STATISTICA 13.3 program (StatSoft Inc., Tulsa, OK, USA).

## 3. Results

The complete cow recovery from serous mastitis occurred after 6 days of Lactobay treatment and after 4 days of Argovit-C treatment.

Preliminary microbiological examination of milk samples from 400 cows with serous mastitis revealed isolates of *S. aureus* in 90% of samples, *S. epidermidis* in 55%, *S. dysgalactiae* in 49.5%, *S. agalactiae* in 45%, *S. pyogenes* in 40% and *E. coli* in 12.7% (Table 1). The present work is dedicated to *S. aureus*. Results of the study of other microorganisms will be published elsewhere.

### 3.1. Antibiotic Susceptibility Changes after Treatments

**Figure 2:**
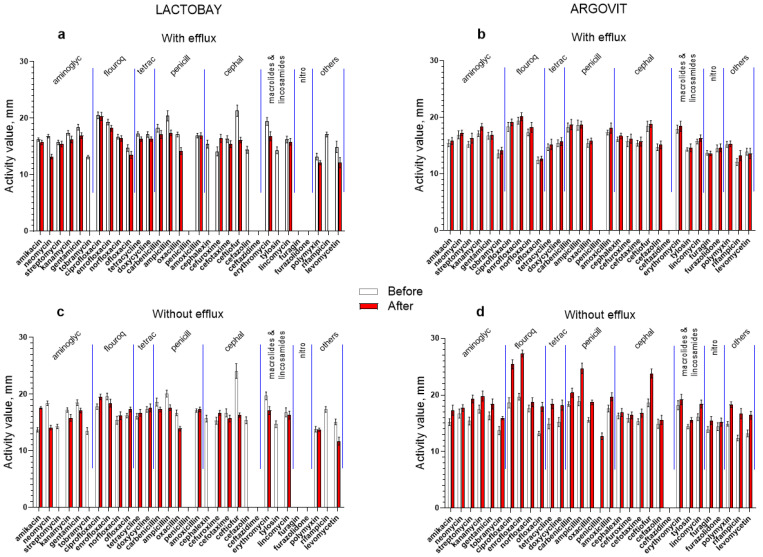
Activity of 31 antibiotics against *S. aureus* before (white columns) and after (red columns) cow treatment with Lactobay (**a**,**c**) and Argovit-C (**b**,**d**) for isolates with the efflux effect (**a**,**b**) and without the efflux effect (**c**,**d**).

**Figure 3:**
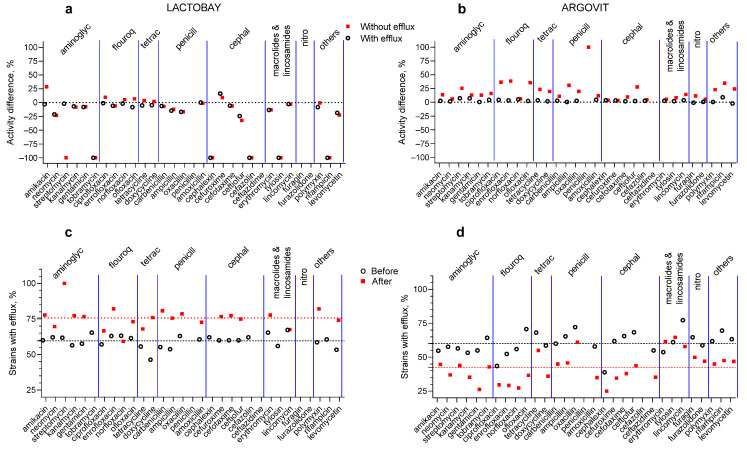
Percentage difference between activity (activity value after treatment minus activity value before treatment) of 31 antibiotics against *S*. *aureus* for treatment with Lactobay (**a**) and Argovit-C (**b**): isolates with the efflux effect (white circles) and without the efflux effect (red squares) and contribution of isolates with the efflux effect before (white circles) and after (red squares) treatment with Lactobay (**c**) and Argovit-C (**d**).

In Figure 2 the results of original data on the activity of 31 antibiotics against *S. aureus* in the samples before and after cow treatment with Lactobay or Argovit-C are presented. Figure 2a,b illustrates the percentage of difference between activity after and before treatment for every antibiotic for both formulations. These results for isolates with and without the efflux effect are presented separately. The most essential difference between the data for Lactobay and Argovit-C consists in the fact that Lactobay treatment led predominantly to a decrease in antibiotic activity, while Argovit-C treatment led to an increase in antibiotic activity (Table 2 and Table 3, Figure 2 and Figure 3).

### 3.2. Lactobay

*S. aureus* were not susceptible (were resistant) in relation to four antibiotics (penicillin, ceftazidime, furagin and furazolidone) before and after Lactobay treatment (Figure 3a). After Lactobay treatment, antibiotic activity towards *S. aureus* decreased by, on average, 25% for both isolates, with and without the efflux effect (Table 2). Additionally, after Lactobay treatment susceptibility to 5 and 6 antibiotics completely disappeared for isolates with and without the efflux effect, respectively (Figure 3a and Table 2). The activity difference is predominantly negative and lies between 0 and −100% (for 45 from 53 experiments) (Figure 3a, Table 2). Between data for isolates with and without the efflux effect some difference was observed. The activity slightly increased after Lactobay treatment (data above 0%) for isolates without the efflux effect for seven antibiotics (mainly for fluoroquinolones and tetracyclines), but for isolates with the efflux effect only for one (cefuroxime) (Figure 3a, Table 2).

### 3.3. Argovit-C

After Argovit-C treatment, activity increased for isolates with and without efflux effects for the vast majority of antibiotics (for 57 from 59 cases), and therefore the activity difference is positive (interval of difference between 0 and 100%, Figure 2b, Table 3). After Argovit-C treatment, antibiotic the activity towards *S. aureus* increased for isolates with efflux by 3% on average and for isolates without the efflux effect by 20% on average. Moreover, Argovit-C treatment led to the appearance of the susceptibility of *Staphylococcus* isolates without the efflux effect to penicillin, which was absent before this treatment. It should also be noted that *S. aureus* was not susceptible in relation to ceftazidime before and after Argovit-C treatment for both isolate types (Figure 2b, Table 3).

### 3.4. Changes in the Portion of Isolates with the Efflux Effect after Treatments

The changes in the portion of isolates with the efflux effect after treatments for Lactobay and Argovit-C are presented in Figure 2c,d, respectively. This portion increased by an average of 15% (from 60 to 75%) after Lactobay treatment and decreased by an average of 17.5% (from 60 to 42.5%) for Argovit-C. There are two exceptions for Lactobay (norfloxacin and lincomycin) and two exceptions for Argovit-C (erythromycin and tylosin). For these four cases, no change or low change in the opposite direction were observed.

## 4. Discussion

After Lactobay treatment, antibiotic activity towards *S. aureus* decreased by, on average, 25% for both isolates, with efflux and without efflux effects. For six antibiotics, the susceptibility was lost. For four antibiotics (penicillin, ceftazidime, furagin and furazolidone), susceptibility was observed neither before nor after treatment. Although it is declared by the manufacturer that Lactobay active components ampicillin and cloxacillin are antibiotics of the penicillin series, the combination of which provides a wide range of bactericidal properties, including against penicillin-resistant bacteria [30], clinical *S. aureus* isolate was resistant to penicillin before and after Lactobay treatment.

After Argovit-C treatment, antibiotic activity towards *S. aureus* increased for isolates with efflux by, on average, only 3% and for isolates without efflux effects by, on average, 20%. Moreover, the susceptibility was not lost as in the case of Lactobay (where susceptibility to six antibiotics was lost completely), but on the contrary, susceptibility to one antibiotic (penicillin) appeared. Activity remained absent for one antibiotic for isolates without efflux and two antibiotics for isolates with the efflux effect (Table 2 and Table 3). therefore, Lactobay treatment resulted in a 25% decrease in *S. aureus* susceptibility to the studied antibiotics, while Argovit-C treatment resulted in increases of 3 and 20% for isolates with and without efflux effects, respectively.

Results presented in Figure 2c,d elucidate the observed difference between the portion of isolates with the efflux effect for Lactobay and Argovit-C treatments. Lactobay treatment led to a 15% increase, while Argovit-C treatment led to a 17.5% decrease in the portion of isolates with capacity to eliminate antibiotics from *S. aureus* bacteria with the efflux effect. It is worth mentioning the following: Despite the fact that after Argovit-C treatment the portion of isolates without the efflux effect and their antibiotic activity significantly increased by 17.5 and 20%, respectively, the antibiotic susceptibility of isolates without the efflux effect increased negligibly (3%), although their portion decreased by 17.5%. Thus, the bacteria that retained the efflux effect after the treatment practically did not increase in susceptibility to antibiotics.

Staphylococci studies suggest that the principal resistance mechanism is the expression of active efflux pumps, which act as the first line of defense against antimicrobials [31]. Below, we briefly describe the efflux mechanism of *S. aureus* in relation to the antibiotic groups used in the present work.

In staphylococci, NorA is the main efflux pump related to the first-line response to antimicrobials [32]. It is overexpressed in 43% of *S. aureus* strains [33]. In *S. aureus*, the plasmid (pSK1)-borne *qac* genes are responsible for aminoglycoside efflux [34]. The efflux mechanism involved in *S. aureus* resistance to fluoroquinolones, quinolones, and tetracycline include the following efflux proteins/pumps [32]. NorA and QacA represent the best studied major facilitator superfamily multidrug efflux pumps found in *S. aureus* [35]. NorA confers resistance to hydrophilic fluoroquinolones antibiotics and a broad range of structurally different compounds [32]. In *S. aureus*, TetA(K) and Tet38 drug efflux pumps confer resistance to the tetracycline antibiotics [32]. Recently novel Tet(L) efflux pump variants were revealed. They confer resistance in *Staphylococcus* spp. to tetracyclines (tigecycline and eravacycline) [36]. In staphylococci, the MsrA pump encoded on plasmids develops resistance to macrolides (only with 14- and 15-membered rings) and to type B streptogramins [37]. The *MsrA* gene encodes a protein with two ATP-binding domains characteristic of ABC transporters [38]. The efflux system seems to be multicomponent, involving *MsrA* and chromosomal genes to create a fully functioning efflux pump [32].

The mechanism of the efflux effect in *S. aureus* as a resistance mechanism developed for nitrofurans, penicillins and cephalosporins has not been studied because: (1) the emergence of resistance to nitrofurans is normally slow [39] and (2) the penicillins and cephalosporins (the β-lactams) act by binding to penicillin-binding proteins and disrupt peptidoglycan cross-linking in the cell wall synthesis, that lead to bacterial lysis and death of the cell [40]. These two last antibiotic groups attack microorganisms from the outside, and therefore the efflux effect loses its importance.

Our experimental results indicate that Argovit-C AgNPs are capable of increasing *S. aureus* susceptibility to antibiotics of different groups. It can be explained at least partially by a decrease in the portion of isolates with the efflux effect after AgNPs treatment revealed in ex vivo experiments of our work. In previous papers, interactions of AgNPs with efflux pump proteins or modulation of efflux pump genes were studied in in vitro experiments. Expression of AcrB inner membrane protein was reduced in *E. cloacae* isolates upon addition of AgNPs that was interpreted as indirect evidence of AgNPs functioning as efflux pump inhibitors (EPIs) [12]. The downregulation of *adeB* and *mexB* membrane proteins was also seen after treatment with AgNPs: multidrug resistant (MDR), and non-MDR *P. aeruginosa*, as well as wild-type and MDR *A. baumanni* [20]. In [16], it was shown that among 50 strains, 12 MDR *A. baumannii* strains possessed efflux pump genes and the expression of *AdeA*, *AdeC*, *AdeS*, *AdeR*, *AdeI*, *AdeJ* and *AdeK* genes was considerably downregulated after the treatment with AgNPs. The authors concluded that the biologically synthesized AgNPs used in [16] exhibit EPIs activity, which may be one of the possible mechanisms of their antibacterial activity against MDR *A. baumannii* strains. AgNPs decreased the number of MDR isolates with efflux activity from 94 to 25% in *Acinetobacter* and from 70 to 57% of MDR isolates of *Pseudomonas* [16]. However, in some cases the opposite effect, the activation of efflux pumps due to the exposure to AgNPs, was observed. For example, the MDR *K. pneumoniae* MGH78578 resistance effect against AgNPs stabilized by lysozyme was observed [20]. In [17], it is reported that the inhibition of efflux pumps led to enhanced sensitivity of *E. coli* to 2,2-dibromo-3-nitrilopropionamide and AgNPs.

There are also earlier published results dedicated to the influence of AgNPs in combination with MDR drugs used for treatment of some specific diseases on the efflux pump protein or efflux effect. These studies already bring us closer to understanding the processes of treatment of particular drug-resistant cells/microorganisms with specific drugs. In [23], the treatment with 75 nm AgNPs sensitized drug-resistant cancer MCF-7/KCR cells (MCF-7 human breast adenocarcinoma cell line was purchased from ATCC) to apoptosis induced by doxorubicin (chemotherapy drug) [23]. This conclusion was made based on the observed inhibition of Pgp transporter activity without downregulating its expression by 75 nm AgNPs. The results of [24] indicated that AgNPs have synergy with fluconazole against planktonic cells of fluconazole-resistant *C. albicans* and biofilms. It was revealed that synergy was due to AgNPs decreasing the efflux bump activity. In vivo experiments showed that fluconazole and AgNPs together considerably decreased the fungal burden, improving the survival of infected mice [24]. Well-known synergetic effects of AgNPs and antibiotics can be partially explained by the reducing effect of AgNPs on efflux pump activity. However, this very interesting topic will be discussed elsewhere.

Table 4 illustrates that the influence of AgNPs on the efflux effect was studied on bacterial strains of the following species:

- *P. aeruginosa:* (1) ciprofloxacin resistant, (2) MDR isolates; (3) MDR and non-MDR;
- *E. coli:* (1) (ATCC 700609), (2) biofilms of melioidosis pathogenic Ceftazidime-resistant (O157:H7), (3) ATCC 25922 and isolates of non-susceptible ofloxacin;
- *A. baumannii:* (1) wild-type and MDR, (2) MDR, (3) 7865 (TNAB) and tigecycline susceptible 8010 (TSAB);
- *K. pneumoniae:* (1) multidrug-resistant MGH78578 and (2) isolates (strain ATCC700603);
- *B. pseudomallei:* (1) (1026b H777 and 316c)
- *E. cloacae:* (1) clinical isolate (EspIMS6) and subsp. cloacae ATCC-13047.

Moreover, the influence of AgNPs on the efflux effect was studied on *Bifidobacterium bifidum* probiotic, the drug-resistant cancer MCF-7/KCR cell line and planktonic cells and biofilms (Table 4). However, we could not find results for the influence of AgNPs on the efflux effect for *Staphylococcus.*

The studies on the influences of other types of nanoparticles (TiO_2_NPs, AuNPs, CuONPs, etc.) on the efflux effect in *P. aeruginosa* in the presence of several antibiotics were also carried out. In [42], it was revealed that the use of TiO_2_NPs alone or in combination with one of four antibiotics (ciprofloxacin, meropenem, amikacin, and ceftriaxone) led to significant downregulation of genes of the efflux pump (*MexY*, *MexB*, *MexA*) and QS-regulated genes (*lasR*, *lasI*, *rhll*, *rhlR*, *pqsA*, *pqsR*) in MDR *P. aeruginosa*. The authors concluded that TiO_2_NPs can increase the therapeutic efficacy of conventional antibiotics by changing efflux pump expression [42]. The authors of [43] with EtBr the agar cartwheel method showed that the CuO, ZnO and CuZn NPs at the sub-minimum inhibitory concentration enhanced the fluorescence, indicating the existence of the efflux effect, while in the absence of NPs, there was a much lower fluorescence. The experiments were undertaken for three drugs (meropenem, ciprofloxacin, and cyanide 3-chlorophenylhydrazone) and MDR clinical strains of *P. aeruginosa* [43]. AuNPs transmitted susceptibility to the last line antibiotics meropenem, trimethoprim, and five widely used antibiotics (levofloxacin, ciprofloxacin, tigecycline, chloramphenicol, and fosfomycin) in *P. aeruginosa* clinical isolates, which had resistance to these antibiotics. A RT-qPCR study of clinically relevant MexAB-OprM efflux pump components demonstrated downregulation in mexB and OprM transcripts in AuNPs-treated *P. aeruginosa* clinical isolates [44].

The literature results described above illustrate that AgNPs alone or in the presence of drugs can inhibit activity of efflux pumps and thereby revive the drug activity, decreasing resistance of microorganisms or cells toward these drugs. According to our literature analysis, these experiments were undertaken in vitro. To the best of our knowledge, our results represent the first systematic in vivo fieldwork carried out in a breeding cow farm. The present work included 400 cows with serous mastitis, studied in triplicates regarding their antibiotic susceptibility to 31 antibiotics before and after two different treatments; the efflux effect was also studied. Thus, this work made a large-scale transition from the research in the laboratory to research under conditions of a livestock farm. As a matter of fact, the present work is a translational study aimed at translating (converting) basic research results into results that directly serve a human benefit. The treatment of mastitis in cows is of great interest to the agro-industrial complex [45]. In the present work, it was revealed that complete cow recovery from serous mastitis occurred after 6 days of Lactobay treatment and after 4 days of Argovit-C treatment. Hence, treatment with Argovit-C resulted as more effective than that with the first-line veterinary medicine Lactobay. Moreover, it is important to mention that Argovit-C since the year 2000 was certified for veterinary application as a drug for gastrointestinal diseases of calves for therapeutic and prophylactic purposes. This will simplify procedures of Argovit-C optimization and certification for mastitis treatment.

The results obtained here reinforce the hope that the future AgNPs application as efflux pump inhibitors could recuperate the bactericidal effect of drug-resistant antibiotics, decrease their dose, and keep wide-spectrum antibiotics on the market. Our results showed that mastitis treatments with Argovit-C AgNPs led to partial restoration of the activity of the majority of 31 studied antibiotics. This allows to suggest that treatments of other diseases with AgNPs could gradually restore (completely or partially) the activity of MDR antibiotics. However, further studies are necessary because obtained results raise a number of important questions. Can 20% susceptibility increase caused by Argovit-C treatment be repeatedly observed after the successive treatments of the same cow group or does this increase happens only once? Does the 20% susceptibility improvement drop with subsequent treatments? Is it possible to increase the effect of antibiotic reactivation more than 20% by varying Argovit-C concentration, its doses, and administration frequency? Will microorganisms due to repeated treatments with AgNPs develop resistance? Is it possible to achieve restoration of susceptibility to antibiotics not only for *S. aureus*, but for other bacteria? Is Argovit-C the only AgNPs formulation that has the property to decrease the antibiotic resistance? Or are there other types of AgNPs formulations that have a similar capacity?

## 5. Conclusions

To the best of our knowledge, the present work represents the first translational research with AgNPs application as an approach for solving the worldwide drug resistance problem. The translational character of the present research is justified because it aimed to translate (converting) basic research results into results that directly serve a human benefit. In vivo fieldwork was carried out with 400 breeding farm cows sick with a serous mastitis. Ex vivo laboratory experiments was undertaken with clinical *S. aureus* isolates sampled from cow breast secretion. They included the study of the efflux effect availability and susceptibility of *S. aureus* isolates to 31 antibiotics of 8 groups before and after intracisternal cow treatment with two veterinary drugs (mixed antibiotic Lactobay and Argovit-C silver nanoparticles).

Argovit-C showed a number of advantages compared with Lactobay, which is the first-line drug for cow mastitis treatment. Lactobay treatment resulted in a 25% decrease in *S. aureus* susceptibility to studied antibiotics, while Argovit-C treatment led to 20 and 3% susceptibility increases (for isolates without and with the efflux effect, respectively). Moreover, the susceptibility to six antibiotics was completely lost after Lactobay treatment, but after Argovit-C treatment, on the contrary, susceptibility to one antibiotic (penicillin) reappeared. Among 31 antibiotics, no susceptibility was observed only for three antibiotics in the case of Argovit-C treatment and for eight antibiotics in the case of Lactobay treatment. Moreover, Lactobay treatment led to a 15% increase, while Argovit-C treatment led to a 17.5% decrease in the portion of isolates with the efflux effect, which resulted in the elimination of antibiotics from *S. aureus*. Obtained results showed that mastitis treatments with Argovit-C AgNPs led to partial restoration of the activity of the majority of the 31 studied antibiotics. This allows to suggest that treatments of other diseases with AgNPs could gradually restore (completely or partially) the activity of MDR antibiotics.

Taking into account that complete recovery from mastitis with Argovit-C treatment lasted 4 days and with Lactobay 6 days, and the fact that Argovit-C is certificated for treatment of cattle gastrointestinal diseases, Argovit-C can be considered as a promising formulation for mastitis treatment without aggravating the antibiotic resistance. Obtained results indicate the necessity of further translational research for other diseases and other AgNPs formulations, which will contribute to solving the worldwide drug resistance problem. The obtained results allow to suggest that disease treatments with AgNPs can gradually restore (completely or partially) the activity of antibiotics to which bacteria have already developed resistance.

## Figures and Tables

**Table 1:** Results of microbiological examination of milk samples from 400 cows with serous mastitis.

Microorganisms	Number of Isolates	%
*S. aureus*	360	90
*S. epidermidis*	220	55
*S. dysgalactiae*	198	49.5
*S. agalactiae*	180	45
*S. pyogenes*	160	40
*E. coli*	51	12.7

**Table 2:** Change in antibiotic activity towards *S. aureus* observed after treatment with Lactobay for isolates with and without the efflux effect.

Activity Change Category	Isolates without the Efflux Effect		Isolates with the Efflux Effect	
	Number of Antibiotics	Average Change in Activity	Number of Antibiotics	Average Change in Activity
Cumulative change				
Total activity change	27	−25.8%	27	−24.5%
Total activity change for 54 samples	−25.1%			
Changes detailed				
Activity remains absent	4	0	4	0
Activity disappeared (−100%)	6	−100%	5	−100%
Activity appeared (+100%)	0	0	0	0
Activity decreased (−Δ%)	14	−11.5%	20	−9.1%
Activity increased (+Δ%)	7	+9.1%	1	+16.0%
Activity constant (0%)	0	0	1	0

**Table 3:** Change in antibiotic activity towards *S. aureus* observed after treatment with Argovit-C for isolates with and without the efflux effect.

Activity Change Category	Isolates without the Efflux Effect		Isolates with the Efflux Effect	
	Number of Antibiotics	Average Change in Activity	Number of Antibiotics	Average Change in Activity
Cumulative change				
Total activity change	30	+19.9%	29	+2.9%
Total activity change for 59 samples	+11.4%			
Changes detailed				
Remain absent	1	0	2	0
Disappeared (−100%)	0	0	0	0
Appeared (+100%)	1	+100%	0	0
Decreased (−Δ%)	0	0	2	−1.4%
Increased (+Δ%)	29	+17.1%	27	+3.2%
Constant (0%)	0	0	0	0

**Table 4:** Influence of AgNPs on the efflux effect in different biological systems.

Biological System	Results Concerning the Efflux Effect	Reference
Ciprofloxacin-resistant *P. aeruginosa*.	AgNPs alone and with ciprofloxacin considerably decreased the expression of bacterial efflux pump *MexA* and *MexB* genes.	[14]
*P. aeruginosa* MDR isolates and *A. baumannii* Clinical antibiotic-resistance isolates	Efflux pump inhibitor activity of AgNPs was observed in 25 and 57% of isolates of *Acinetobacter* and *Pseudomonas*, respectively.	[15]
MDR, and non-MDR *P. aeruginosa* and wild-type and MDR *A. baumannii*	AgNPs had efflux pump inhibitor activity against *A. baumannii* and *P. aeruginosa*.	[20]
Multidrug-resistant *A. baumannii*	*AdeA*, *AdeC*, *AdeS*, *AdeR*, *AdeI*, *AdeJ*, and *AdeK* genes were downregulated considerably after AgNPs treatment.	[16]
*E. coli* (ATCC 700609)	Efflux pump inhibition led to enhancement of sensitivity to two antimicrobials.	[17]
Biofilms of melioidosis pathogenic Ceftazidime-resistant *E. coli* (O157:H7) and *B. pseudomallei* (1026b H777 and 316c)	AgNPs continue to exhibit a strong efflux pump inhibition against *B. pseudomallei* even after extended exposure for 30 passages with sublethal doses.	[19]
*E. coli* ATCC 25922, isolates of ofloxacin non-susceptible *E. coli* (N-*E. coli*), tigecycline non-susceptible *A. baumannii* 7865 (TNAB), and tigecycline susceptible *A. baumannii* 8010 (TSAB)	Surfactant modified AgNPs could decline the activity of efflux pumps AdeABC and AdeIJK in drug-resistant *A. baumannii* due to inhibition of the efflux pump genes *ade B* and *ade J*, in tigecycline-susceptible *A. baumannii* 8010 (TSAB).	[41]
Multidrug-resistant *K. pneumoniae* MGH78578	Activation of efflux pumps after exposure to AgNPs.	[20]
*K. pneumoniae* isolates (strain ATCC700603)	Biosynthesized AgNPs reduce the expression of OxqAB efflux pump genes.	[21]
*E. cloacae* clinical isolate (EspIMS6) and *E. cloacae* subsp. cloacae ATCC-13047	AgNPs addition reduced expression of functional AcrB protein in *E. cloacae*.	[12]
*Bifidobacterium bifidum* (probiotic)	Both biosynthesized and commercial AgNPs decreased the *oxqAB* gene expression levels.	[21]
The drug-resistant cancer MCF-7/KCR cell line (purchased from ATCC) was developed from MCF-7 under Doxorubicin (chemotherapy drug) in selection pressure from 10 nM to 1 μM	75 nm AgNPs considerably inhibited P-glycoprotein efflux activity in DR breast cancer cells, while 5 nm AgNPs did not.	[23]
Planktonic cells and biofilms	AgNPs downregulated *ERG1*, *ERG11*, *ERG25* and *CDR2*, decreased levels of membrane ergosterol and membrane fluidity, decreased membrane content of Cdr1p, Cdr2p, and consequently efflux pump activity.	[24]

## Data Availability

Available by request to the corresponding author.
